# Sense of coherence as a predictor of onset of depression among Japanese workers: a cohort study

**DOI:** 10.1186/1471-2458-11-205

**Published:** 2011-04-01

**Authors:** Toshimi Sairenchi, Yasuo Haruyama, Yumiko Ishikawa, Keiko Wada, Kazumoto Kimura, Takashi Muto

**Affiliations:** 1 Department of Public Health, Dokkyo Medical University School of Medicine, Shimotsugagun-Mibu, Japan; 2 Dokkyo Medical University School of Nursing, Shimotsugagun-Mibu, Japan; 3 Center for Medical Informatics, Dokkyo Medical University Hospital, Shimotsugagun-Mibu, Japan

## Abstract

**Background:**

The ability to predict future onset of depression is required for primary prevention of depression. Many cross-sectional studies have reported a correlation between sense of coherence (SOC) and the presence of depressive symptoms. However, it is unclear whether SOC can predict future onset of depression. Therefore, whether measures to prevent onset of depression are needed in for persons with low SOC is uncertain. Thus, the aim of this cohort study was to determine whether SOC could predict onset of depression and to assess the need for measures to prevent onset of depression for persons with low SOC.

**Methods:**

A total of 1854 Japanese workers aged 20-70 years in 2005 who completed a sense of coherence (SOC) questionnaire were followed-up until August 2007 using their sick-pay records with medical certificates. Depression was defined as a description of "depression" or "depressive" as a reason for sick leave on the medical certificates. The day of incidence of depression was defined as the first day of the sick leave. Risk ratios of SOC for onset of depression were calculated using a multivariate Cox proportional hazards model.

**Results:**

Of the 1854 participants, 14 developed depression during a mean of 1.8 years of follow-up. After adjustment for gender and age, the risk ratio of high SOC compared with low SOC for sick leave from depression was 0.18 (95% confidence interval [CI], 0.04 to 0.79). The area under the receiver operating characteristic curve of SOC was 0.70 (95% CI, 0.58 to 0.82).

**Conclusions:**

The SOC may be able to predict onset of depression in Japanese workers. Measures to prevent onset of depression for persons with low SOC might be required in Japanese workplaces. Thus, SOC could be useful for identifying persons at high risk for future depression.

## Background

Depression affects not only a worker's well-being and productivity but also increases the risk of suicide[1,2]. The mortality rate from suicide and self-inflicted injury per 100 000 population was 17.1 among men and 4.0 among women in the United States in 2000[3]. The mortality rate from suicide and self-inflicted injury was 35.2/100 000 among men and 12.8/100 000 among women, which was approximately 3-fold higher than the mortality rate from motor vehicle traffic accidents (11.5/100 000 among men and 5.1/100 000 among women) in Japan in 2002[3]. Depression is, therefore, an important public health issue. Primary prevention of depression requires the ability to predict future onset of depression.

Antonovsky constructed a concept called "sense of coherence (SOC)" and showed that SOC was inversely associated with state anxiety response[4]. In addition, many studies showed that SOC was strongly related to health[5]. Previous cross-sectional studies have reported a significant inverse correlation between SOC and depression[6-9]. A cross-sectional study showed that SOC was inversely related to Beck's Depression Inventory (BDI) and Beck's Anxiety Inventory (BAI) in adolescent females aged 15.9-17.7 years in Stockholm, Sweden[6]. A nationwide study of 2003 Swedish men and women with a mean age of 44.2 years, showed an inverse correlation between SOC and depressive symptoms[7]. A controlled study of South Africans aged 17-69 years showed an inverse correlation between SOC and BDI[9]. Furthermore, a study of 50 Japanese patients with systemic sclerosis aged 33-75 years showed an inverse correlation between SOC and BDI[8].

Some possible mechanisms to explain the relationship between SOC and depression have been identified. Many previous studies have reported the stress-buffering effects of a strong SOC[10]. A strong SOC was related to a lower rating of stress for given life events[11], fewer reports of having stressful events[12], less emotional distress[13], and a lower level of anxiety[4,14,15]. Based on these previous studies, a causal relationship between SOC and depression has been hypothesized. Nevertheless, the temporality of the relationship between SOC and depression has not been ascertained because the previous studies were cross-sectional. In contrast, a cohort study showed that SOC significantly predicted any sickness absences, which might have included depression, in female employees aged 20-56 years in Raisio, Finland[16].

The results of these previous studies lead to the hypothesis that SOC can predict future onset of depression. However, to the best of our knowledge, limited data are available for the relationship between SOC and the future onset of depression. Therefore, whether measures to prevent onset of depression are needed for persons with low SOC is uncertain. Thus, a cohort study was conducted to examine whether SOC could predict future onset of depression and to clarify whether measures to prevent onset of depression are needed for persons with low SOC.

## Methods

### Participants

**Figure 1:**
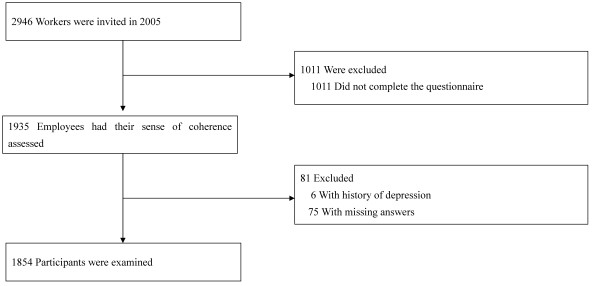
A detailed flow diagram of the study participants

In the present cohort study, 2946 workers aged 20-72 years who worked for a software development company in Tokyo, Japan were invited to undergo a mental health questionnaire that was conducted by the Healthcare Marketing Intelligence Incorporated Company in September 2005 (Figure 1). A total of 1011 workers did not complete the questionnaire. Of the 1935 workers who completed the questionnaire (response rate, 65.7%), 6 workers with a history of depression and 75 workers with incomplete questionnaires were excluded. Thus, 1854 participants (1395 men and 459 women), aged 20-70 years in 2005, were followed-up until August 2007 using their sick-pay records.

Data were collected from the Healthcare Marketing Intelligence Incorporated Company with complete anonymity. The protocol of this cohort study was approved by the institutional review board of Dokkyo Medical University School of Medicine.

### Measurements of Sense of Coherence

The self-rated questionnaire consisted of questions about gender, age (years), administrative post (yes or no), married (yes or no), and SOC. The SOC was measured by 13 questions that were answered using a 5-point Likert scale. The Japanese version of the SOC, 13-item, 5-point questionnaire was developed by Yamazaki[17]. The validity of this questionnaire has been examined[18].

### Definition of Depression

The participants were followed-up using their sick-pay records with medical certificates. When a worker needs sick leave that is longer than three days, sick pay is paid to the worker by Japanese health insurance. A medical certificate is needed to apply for sick pay. In the present study, the sick-pay records with medical certificates were linked to the mental health questionnaires by the workers' insurance numbers. Depression was defined as a description of "depression" or "depressive" as a reason for sick leave on the medical certificates. The day of incidence of depression was defined as the first day of the sick leave.

### Statistical Analysis

The participants were divided according to the median (low and high) based on the total SOC at baseline. The participants were also divided according to the median for each subscale (meaningfulness, manageability, and comprehensibility) at baseline.

Risk ratios of the total SOC and the subscales for onset of depression were calculated using a Cox proportional hazards model. Person-years of follow-up were calculated from September 1st, 2005 to the date of layoff due to depression, resignation, or August 1st, 2007, whichever occurred first. Gender was included in the model as a covariate (model 1). The analysis was repeated with age (years) (model 2), administrative post (yes or no) (model 3), and married (yes or no) (model 4), respectively, as a covariate in addition to gender.

The sensitivities and specificities of cut-off points for total SOC were calculated. The areas under the curve (AUCs) of the receiver operating characteristic (ROC) curves of the total SOC and the subscales for onset of depression were also calculated.

The statistical analysis, except the sensitivity, specificity, and AUC, were performed using SAS, version 9.1 (SAS Institute, Inc., Cary, NC, USA). The statistical analysis of sensitivity, specificity, and AUC was conducted using Dr. SPSS II (SPSS Inc., Chicago, IL, USA).

## Results

The baseline characteristics of the study participants according to total SOC category are shown in Table 1. The proportion of male participants was significantly higher in the high SOC category than in the low SOC category. The mean age was significantly older in the high SOC category than in the low SOC category. The proportion of participants with administrative posts was significantly higher in the high SOC category than in the low SOC category. The proportion of married participants was significantly higher in the high SOC category than in the low SOC category. No significant difference in working hours per day was found between the SOC categories.

**Table 1:** Baseline characteristics of the 1854 Japanese workers in 2005

	Score for sense of coherence (range)		p for difference*
		
	Low (16-39)	High (40-64)	
Number of participants	954	900	
Male, n (%)	698 (73.2)	697 (77.4)	0.033
Age, mean ± SD (years)	34.7 ± 8.1	36.5 ± 8.9	<0.0001
Administrative post, n (%)	357 (37.4)	425 (47.2)	<0.0001
Married, n (%)	413 (43.3)	503 (55.9)	<0.0001
Working hours per day, mean ± SD	9.3 ± 1.5	9.3 ± 1.4	0.503

*Tested by t-test for age and working hours per day, and by χ2 test for gender, administrative post, and married status.

Of the 1854 participants, 14 developed depression during a mean of 1.8 years of follow-up. The median sick leave of the 14 participants was 30 days (range, 14 to 94 days). Table 2 shows the risk ratios of the total SOC and the subscales for onset of depression. Compared with low total SOC, the gender-adjusted risk ratio of high total SOC was 0.17 (95% confidence interval [CI], 0.04 to 0.76), which was statistically significant. Similarly, the gender- and age-adjusted risk ratio of high SOC was 0.18 (95% CI, 0.04 to 0.79), which was statistically significant. The gender- and administrative post-adjusted risk ratio of high SOC was 0.18 (95% CI, 0.04 to 0.79), which was statistically significant. The gender- and married-adjusted risk ratio of high SOC was 0.18 (95% CI, 0.04 to 0.81), which was statistically significant. No significant association was found between risk for onset of depression and the SOC subscales.

**Table 2:** Risk ratios for layoff for depression by score for sense of coherence in 1854 Japanese workers in 2005-2007

Score for sense of coherence	No. of participants	Person-years	No. of layoffs for depression	Layoff rate per 1,000 person-years	Model 1*	Model 2†	Model 3‡	Model 4§
Total sense of coherence								
Low	954	1699.2	12	7.1	1.00	1.00	1.00	1.00
High	900	1645.1	2	1.2	0.17 (0.04-0.76)	0.18 (0.04-0.79)	0.18 (0.04-0.79)	0.18 (0.04-0.81)
Meaningfulness								
Low	873	1560.9	9	5.8	1.00	1.00	1.00	1.00
High	981	1783.4	5	2.8	0.49 (0.17-1.47)	0.51 (0.17-1.53)	0.51 (0.17-1.52)	0.54 (0.18-1.63)
Manageability								
Low	868	1552.1	8	5.2	1.00	1.00	1.00	1.00
High	986	1792.2	6	3.3	0.64 (0.22-1.85)	0.64 (0.22-1.85)	0.65 (0.23-1.89)	0.66 (0.23-1.91)
Comprehensibility								
Low	1012	1804.5	11	6.1	1.00	1.00	1.00	1.00
High	842	1539.8	3	1.9	0.31 (0.09-1.10)	0.32 (0.09-1.15)	0.32 (0.09-1.14)	0.32 (0.09-1.15)

*Adjusted for gender. †Adjusted for gender and age. ‡Adjusted for gender and administrative post. §Adjusted for gender and married status.

The AUC of the total SOC was 0.70 (95% CI, 0.58 to 0.82), which was statistically significant (p = 0.010) (Table 3). Similarly, the AUC for comprehensibility was 0.70 (95% CI, 0.57 to 0.82), which was statistically significant (p = 0.011). On the other hand, the AUCs for meaningfulness and manageability were not significant.

**Table 3:** Area under the curve for the sense of coherence and its subscales

Subset of sense of coherence	Area under the curve	(95% CI)			p†
Total sense of coherence	0.70	0.58	-	0.82	0.010
Meaningfulness	0.65	0.49	-	0.81	0.057
Manageability	0.65	0.50	-	0.80	0.053
Comprehensibility	0.70	0.57	-	0.82	0.011

*Under non-parametric assumption. †p for H_0_: area under the curve = 0.5.

## Discussion

The present prospective cohort study showed that high SOC was associated with an approximately 80% avoidance of risk for sick leave from depression among Japanese workers. To the best of our knowledge, this is the first cohort study that showed an association between SOC and risk of future onset of depression. Our results suggest that there may be a need for measures to prevent onset of depression for persons with low SOC.

Many cross-sectional studies have reported a significant inverse correlation between SOC and depression[6-9]. A cross-sectional study showed that SOC was inversely related to BDI and BAI in adolescent females aged 15.9-17.7 years in Stockholm, Sweden[6]. A nationwide Swedish study[7], involving 2003 Swedes (976 men and 1027 women) with a mean age of 44.2 years, showed an inverse correlation between SOC (13-item version) and low mood/depressive symptoms. A controlled study of 50 patients aged 17-57 years with major depression and 50 control subjects aged 18-69 years showed an inverse correlation between SOC and BDI in South Africa[9]. A study of 50 Japanese patients with systemic sclerosis aged 33-75 years showed an inverse correlation between SOC (13-item version) and BDI (r = -0.543)[8]. These previous results are consistent with those of the present study.

In contrast, a longitudinal study of 402 mass-evacuated adults from Kosovo reported that SOC at baseline could not predict the diagnosis or symptoms of depression 1.5 years after the baseline survey, while the SOC correlated with symptoms of depression at baseline[19]. The participants of the previous study were not general workers, as were the participants of the present study. In addition, the sample size of the previous study was smaller than that of the present study.

The present study also showed significant AUCs of total SOC and comprehensibility for risk of depression. The results suggest that low comprehensibility was the best predictor among the three subscales of SOC, as well as total SOC. However, the previous cross-sectional study[9], mentioned above, reported that low meaningfulness correlated with BDI in both the depressed group and the control group. Reasons for the inconsistency with the present study are uncertain. SOC may differ in its ability to predict forthcoming depression and screen for present depression.

Possible mechanisms behind the association between SOC and onset of depression can be explained in several ways. A strong SOC has been shown to be related to a lower rating of stress for given life events[11], fewer reports of having stressful events[12], less emotional distress[13], and a lower level of anxiety[4,14,15].

The strength of the present study comes from the use of a medical certificate for ascertainment of onset of depression during the follow-up period, as opposed to previous studies which ascertained it using a self-administered questionnaire[7-9].

The present study, however, had several limitations. First, a 5-point Likert scale was used to assess SOC instead of Antonovsky's 7-point Likert scale. Measurement using the 5-point scale is common in Japan, because the 7-point scale may lead to individual differences in the distribution of the evaluation compared with the 5-point scale[18]. Thus, the absolute SOC results in the present study were not comparable with those of previous studies. However, this limitation does not negate the relationship between SOC and the risk of depression. Second, workers may hesitate to visit clinics for mental issues. Consequently, the hesitation might lead to underestimation of the diagnosis of depression. Third, the diagnosis of depression was not made according to the Diagnostic and Statistical Manual of Mental Disorders, 4th edition (DSM-IV). A nested case-control study[20] that involved 87 Finnish workers with serious burnout and 87 workers without burnout showed that SOC at baseline predicted serious burnout 10 years after the baseline. Therefore, the diagnosis of depression in the present study might include burnout. However, burnout has been reported as a mediator of the association between job strain and depression[21]. Fourth, several possible confounding factors, such as socioeconomic status, could not be adjusted for in the present analysis. In Japan, however, socioeconomic status, such as income, seems to strongly correlate with age, which was adjusted in the present study, in each company. Finally, generalizability is uncertain, since all study participants worked in the same company. Furthermore, the psychological characteristics of Japanese subjects might affect the relationship between SOC and onset of depression. Therefore, our findings may not be generalized to foreign countries. On the other hand, the nested case-control study in Finland showed that SOC at baseline predicted serious burnout 10 years after the baseline[20]. Further studies are warranted to examine the generalizability of these results.

## Conclusions

The SOC may be able to predict onset of depression in Japanese workers. Measures to prevent onset of depression for persons with low SOC may be needed in Japanese workplaces. Thus, SOC could be useful for identifying persons at high risk for future depression.

## Competing interests

The authors declare that they have no competing interests.

## Authors' contributions

TS designed the study, analyzed data, and wrote the final report with all authors contributing to the editing. YH helped develop the analytic strategy. YI wrote the application to the institutional review board. KW helped conduct the literature review. TM supervised the study. All authors read and approved the final manuscript.

## Pre-publication history

The pre-publication history for this paper can be accessed here:

http://www.biomedcentral.com/1471-2458/11/205/prepub
